# Comparing the Mental Health of Healthcare Students: Mental Health Shame and Self-compassion in Counselling, Occupational Therapy, Nursing and Social Work Students

**DOI:** 10.1007/s11469-023-01018-w

**Published:** 2023-02-13

**Authors:** Yasuhiro Kotera, Jessica E. Jackson, Ann Kirkman, Ann-Marie Edwards, Rory Colman, Ann Underhill, Jessica G. Jackson, Denise Baker, Akihiko Ozaki

**Affiliations:** 1 Faculty of Medicine and Health Sciences, Institute of Mental Health, University of Nottingham, Nottingham, NG7 2TU Nottinghamshire UK; 2 College of Health, Psychology and Social Care, University of Derby, Derby, UK; 3 Medical Governance Research Institute, Tokyo, Japan; 4 Department of Breast Surgery, Jyoban Hospital of Tokiwa Foundation, Fukushima, Japan

**Keywords:** Mental health, Healthcare students, Mental health shame, Self-compassion, Attitudes towards mental health problems

## Abstract

Poor mental health of healthcare students is a cause for concern in many universities. Though previous research has identified mental health shame and self-compassion as critical in this student group, how these variables differ across different healthcare disciplines remains to be evaluated. Healthcare students (*n* = 344; counselling, occupational therapy, social work and nursing) completed measures regarding these variables. MANOVA and regression analyses were performed. (1) Counselling and nursing students were more depressed than occupational therapy students; (2) nursing students were more anxious than occupational therapy and social work students; (3) occupational therapy students had more positive attitudes towards mental health than the others; and (4) nursing students worried about their own reputation associated with their family more than counselling students. Self-compassion was the strongest predictor of mental health in all groups; however, the effect sizes varied: largest in nursing and smallest in social work students. Findings will help inform effective interventions for students in each healthcare discipline.

## Poor Mental Health in Healthcare Students

Maintaining a high level of mental health is essential for many, including university students (Kotera et al., 2019a; Kotera et al., 2021g). Good student mental health is associated with a wide range of positive outcomes including better academic performance (Andrews & Wilding, 2004), reduced dropout rates (Hjorth et al., 2016) and higher academic engagement (Kotera & Ting, 2019). However, an increased number of studies have found that students are experiencing poor mental health, with high levels of anxiety, depression and psychotic disorders (Islam et al., 2020; Kotera & Maughan, 2020). Additionally, students have reported increased fatigue, insomnia, difficulty in concentrating, forgetfulness and irritability (Costa et al., 2010). The number of students with psychological issues is rising every year (Zeng et al., 2019).

Among diverse disciplines, students in healthcare subjects such as counselling, occupational therapy, social work and nursing are reported as a highly concerning student population for mental health. High levels of stress, anxiety and depression have been reported by healthcare students (Alzayyat & Al‐Gamal, 2016). Nearly half of nursing students (48%) reported a high level of stress (Nolan & Ryan, 2008). More than a third of social work students (34%) suffered from high levels of depressive symptoms (Horton et al., 2009). Mental distress was reported by one in five nursing students (Nerdrum et al., 2009). Similarly, more than one third of counselling, occupational therapy and social work students reported a high level of depression and low self-esteem (Boellinghaus et al., 2013; Horton et al., 2009). Unsurprisingly, healthcare students are more mentally distressed than the others, and their mental health deteriorates throughout the course (Dyrbye et al., 2006). Several reasons have been identified. Healthcare students are required to go on placements, which account for 50% of their time in the course (Henderson, 2011). Healthcare placements can yield considerable demands on students, negatively impacting their mental health (Clements et al., 2016). High levels of anxiety and stress were reported by healthcare students around placements (Timmins et al., 2011). Accordingly, the numbers of healthcare students who withdraw from the course and take a study break have been increasing in many countries (Hamshire et al., 2019). In the UK, for example, one in three nursing students dropped out in 2020 (Jones-Berry, 2021), and more than half considered leaving the nursing course (Hamshire et al., 2013). These rates are alarmingly high compared with a 7.6% dropout among all undergraduate students in the UK in 2019 (Higher Education Statistics Agency, 2021).

These mental health challenges in healthcare have been exacerbated during COVID-19 (Greenberg et al., 2020). Healthcare workers and students are at the frontline for diagnosing, treating and caring for patients (Rothe et al., 2020), affecting their mental health more negatively than their physical health (Adams & Walls, 2020). In China, 71.5% of healthcare workers reported a high level of mental distress followed by depression (50.4%), anxiety (44.6%) and insomnia (34.0%) (Lai et al., 2020). In a comparison with the general public, healthcare workers had poorer mental health and lower levels of positive psychological attributes (Kotera et al., 2021e). Common stressors are the increasing numbers of COVID-19 cases, overwhelming workload, stigma associated with COVID-19 infection, lack of personal protection equipment and lack of information such as practice guidelines and legal protections (Godlee, 2020). Their usual coping strategies are also prohibited such as visiting family members or friends, or having a social gathering with colleagues (Kotera et al., 2022c). Healthcare workplaces have become a highly stressful setting, which can yield additional stress for healthcare students on placement (Kotera et al., 2021f), who need to balance their clinical practice and academic work (Smith, 2020).

Healthcare students have been experiencing higher levels of anxiety and depression since the outbreak of COVID-19 (Chandratre, 2020). The rates of healthcare students who suffer from mental health symptoms are high: depression (74.6%), insomnia (62.6%) and anxiety (62.3%) (Essangri et al., 2021; Hofstra et al., 2021). Online learning is difficult for healthcare students as their curriculum requires practical training, which can be stressful to be offered virtually (Nishimura et al., 2021; Suzuki et al., 2022). During COVID-19, the university’s support services are closed, which compromised students’ mental health coping (Smith, 2020). Despite these concerns, effective solutions for poor mental health in healthcare students remain to be identified (Muller et al., 2020).

## Shame as Negative Mental Health Factor

Shame can exacerbate mental health problems (Kotera et al., 2019b, 2019c, 2019d) as it elicits strong negative emotions, leading to poor mental health (Yakeley, 2018). Shame can negatively impact our identity (Monroe, 2009) and acceptability in social contexts (Dolezal & Lyons, 2017). Moreover, shame causes us to focus our attention inward and see ourselves negatively, related to self-criticism (Gilbert, 2010). In particular, shame for mental health problems (i.e. mental health shame), believing that mental health problems equate to weakness and inadequacy (Kotera et al., 2022d), has been reported as strongly associated with poor mental health (Kotera et al., 2020b; Kotera et al., 2018, 2019a).

In addition to these negative emotions, shame towards mental health problems has diverse adverse effects on mental health. Mental health shame is often a barrier to seeking help or revealing their mental health issues to others, making their mental health problems worse and increasingly difficult to manage (Hofstra et al., 2021). University students in particular demonstrate a high level of mental health shame, believing that having a mental health problem will cause them to be excluded from the community (Gilbert et al., 2007; Hofstra et al., 2021). Among university students, healthcare students demonstrated a high level of mental health shame as they are trained to offer care for others but not to ask for it (Kotera et al., 2021c). Indeed, self-criticism was identified as a moderator for the mental health shame-mental health problems pathway: mental health shame’s impact on mental health problems was intensified as self-criticism increased (Kotera et al., 2020a). Mental health shame is categorised into negative attitudes (believing mental health problems as a sign of inadequacy), external shame (believing others see mental health problems negatively), internal shame (one sees themselves negatively) and reflected shame (worries for their/others’ reputation because of others’/their mental health problems) (Gilbert et al., 2007), and healthcare students demonstrated a high level of these shame components (Kotera et al., 2019a). Despite the relevance of mental health shame to mental health in healthcare students, how this negative mental health factor differs among students in different healthcare disciplines and relationships with mental health problems remain to be evaluated.

## Self-compassion as Positive Mental Health Factor

A growing body of research demonstrates that practising self-compassion improves mental health (Kotera & Van Gordon, 2021). Self-compassion is a key factor in how people deal with stressful situations, regulating our emotions in a healthful way, without derailment (Bakker et al., 2019; Colman et al., 2022). Self-compassion comprises three elements: (1) treating yourself with kindness rather than being critical towards oneself during times of stress (self-kindness versus self-judgment); (2) recognising our pain and suffering, no matter how painful, is part of the human experience (humanity versus isolation); (3) allowing ourselves to experience feelings without exaggerating or avoiding our emotions (mindfulness versus over-identification) (Neff & Germer, 2022). Self-compassion has been receiving increasing attention in research and practice, as it has been identified as a protective factor for mental health (Kotera & Van Gordon, 2021). Students high in self‐compassion treat themselves with kindness and concern, utilising coping skills effectively (Allen & Leary, 2010). Self-compassion helps healthcare students with mental illnesses by increasing resilience and reducing self-criticism and shame (Kotera et al., 2021a, 2021b). Studies report that students who are self-compassionate tend to have a low degree of mental health shame (Kotera et al., 2022a; Kotera et al., 2022b). Moreover, self-compassion is associated with a natural desire to learn (Neff et al., 2007) and positive mental health outcomes such as reduced stress, depression, anxiety and higher life satisfaction (López et al., 2018). Despite the great impact of self-compassion on mental health in healthcare students, this positive construct remains to be examined across different healthcare student groups.

## Study Aims

Taken together, the present study aimed to evaluate the levels of and relationships among mental health constructs in different healthcare students, in order to offer more tailored insights into the mental health of students in each discipline. Counselling, occupational therapy, social work and nursing students were recruited for their serious mental health concerns. First, we compared the levels of mental health problems, namely depression, anxiety and stress (as these are common in university students (Ramón-Arbués et al., 2020)), mental health shame and self-compassion. Then, we identified significant predictors of mental health problems in each group, and compared the effect sizes. Our research questions were as follows:

RQ1: How different are the levels of mental health problems, mental health shame and self-compassion among counselling, occupational therapy, social work and nursing students?

RQ2: How different the effect sizes of predictors of mental health problems are, among the four groups (counselling, occupational therapy, social work and nursing students)?

## Materials and Methods

### Sample Selection

All participating students were at least 18 years old and studying in a healthcare curriculum at a university in the UK. Students who were on a study break were excluded. Participants were recruited using convenient sampling through hard copy questionnaires distributed by programme tutors instead of the researchers to avoid response biases. Of 470 students approached, 344 students (73% response; 307 females, 37 males; age, *M* = 28.64, SD = 8.83, range 18–58 years; 301 undergraduates and 43 postgraduates) completed three self-report measures regarding mental health problems, mental health shame and self-compassion. Demographics of our sample were similar to the general student populations in healthcare disciplines in the UK (Brown, 2017) (Grant, 2004; Office for Students, 2020). No compensation was offered for participation. Informed consent was received from all students participating in the study. Academic staff who were trained in student well-being were present at the research site, and available mental health support was informed to students prior to the study, to ensure students’ safety. Reasons for withdrawal were not asked of students per ethical guidelines, which had been informed to students before the study. Ethical approval was granted from the University Research Ethics Committee.

### Materials

The Depression Anxiety and Stress Scale (DASS21), a shortened form of DASS42 (Lovibond & Lovibond, 1995), was used to measure mental health problems—depression, anxiety and stress. DASS21 consists of 21 items with a 4-point Likert scale (0—did not apply to me at all to 3—applied to me very much or most of the time), divided into three subscales of seven items: depression (e.g. ‘I couldn’t seem to experience any positive feeling at all’), anxiety (e.g. ‘I was aware of dryness of my mouth’) and stress (e.g. ‘I found it hard to wind down’). Internal consistency of each subscale was high in our sample (*α* = 0.86–0.90).

Mental health shame was assessed using the Attitudes Towards Mental Health Problems scale (ATMHPS) (Gilbert et al., 2007). ATMHPS consists of 35 items on 4-point Likert scale (0—do not agree at all to 3—completely agree), considering seven subscales: negative attitudes of community (1) and family (2); external shame of community (3) and family (4); internal shame (5); family-reflected shame (6); and self-reflected shame (7). Negative attitudes (1, 2) consider how their community and family perceive mental health problems (e.g. ‘My community/family sees mental health problems as something to keep secret’). External shame (3, 4) pertains to how students feel their community and family would see them if they had a mental health problem (e.g. ‘I think my community/family would look down on me’). Internal shame (5) appraises how they regard themselves if they had a mental health problem (e.g. ‘I would see myself as inferior’). Lastly, their reflected shame (6, 7) evaluates their family-reflected shame (how their family would be seen if they had a mental health problem; e.g. ‘My family would be seen as inferior’) and self-reflected shame (worries about their reputation, for a close relative having a mental health problem; e.g. ‘I would worry that others will look down on me’). Internal consistency for all subscales was high (*α* = 0.84–0.94).

Self-Compassion Scale-Short Form (SCS-SF) (Raes et al., 2011), a shortened version of the Self-Compassion Scale (Neff, 2003), was used to measure self-compassion. SCS-SF entails twelve 5-point Likert items (1—almost never to 5—almost always) including ‘I try to be understanding and patient towards those aspects of my personality I don’t like’. Scores are reversed for the items assessing the negative components (1, 4, 8, 9, 11 and 12). Our sample’s internal consistency was high (*α* = 0.72).

### Procedure

All data collected were first screened for outliers and the assumptions for parametric tests. Second, a one-way multivariate analysis of variance (MANOVA) was conducted to evaluate the level differences of mental health problems, mental health shame and self-compassion among counselling, occupational therapy, social work and nursing students. Third, regression analyses were used to identify significant predictors of mental health problems, and their effect sizes. All analyses were processed using IBM SPSS version 27.

## Results

### Comparing the Levels of Mental Health Constructs

No outliers were detected. The assumption of normality and linearity of variables was maintained. A one-way MANOVA was performed to compare the levels of mental health constructs among different healthcare disciplines. Data are expressed as mean ± standard deviation in Table 1. There was a statistically significant difference between the disciplines on the combined variables (mental health problems, mental health shame and self-compassion), *F*(33, 972.95) = 2.68, *p* < 0.0005; Wilks’ Λ = 0.77; partial *η*^2^ = 0.08. Significant differences were found in depression (*F*(3, 340) = 4.57, *p* = 0.004, partial *η*^2^ = 0.04), anxiety (*F*(3, 340) = 3.48, *p* = 0.02, partial *η*^2^ = 0.03), community attitudes (*F*(3, 340) = 9.15, *p* < 0.001, partial *η*^2^ = 0.08) and self-reflected shame (*F*(3, 340) = 3.64, *p* = 0.01, partial *η*^2^ = 0.03).

**Table 1:** Descriptive statistics and significant differences in mental health problems, mental health shame and self-compassion among counselling, occupational therapy, nursing and social work students

Scale	Construct (range)	Counselling		Occupational therapy		Nursing		Social work	
		M	SD	M	SD	M	SD	M	SD
Depression, Anxiety and Stress Scale-21	Depression (0–42)	13.65^a^	11.15	9.22^a, b^	8.80	13.58^b^	11.98	10.11	6.85
	Anxiety (0–42)	11.45	10.02	10.34^c^	7.98	14.22^c, d^	11.03	10.47^d^	8.20
	Stress (0–42)	17.51	10.46	16.22	10.34	19.02	12.35	15.65	8.38
Attitudes Towards Mental Health Problems Scale	Community attitudes (0–12)	4.38^e^	3.08	2.81^e, f, g^	2.72	4.82^f^	3.25	5.11^ g^	3.01
	Family attitudes (0–12)	2.23	3.33	1.75	2.68	2.62	3.56	2.65	2.81
	Community external shame (0–15)	5.67	4.50	4.60	4.35	5.95	4.86	5.34	3.77
	Family external shame (0–15)	2.07	3.58	2.08	3.07	3.12	4.69	2.19	2.96
	Internal shame (0–15)	7.39	5.36	7.50	4.63	7.81	5.08	6.40	4.22
	Family-reflected shame (0–21)	5.71	5.50	6.29	5.02	6.95	5.92	5.52	4.94
	Self-reflected shame (0–15)	2.30^ h^	3.68	3.19	4.18	4.38^ h^	4.79	3.10	3.80
Self-Compassion Scale-Short Form	Self-compassion (1–5)	2.92	0.69	2.84	0.66	2.66	0.69	2.78	0.67

*M*, mean; *SD*, standard deviation. Superscripts^a–h^ indicate significant difference between the two. For example, Superscript ‘a’ means that the mean score of depression was significantly higher in the counselling students than the occupational therapy students

Tukey post hoc tests revealed that for depression scores, counselling students and nursing students had a higher level than occupational therapy students (*p* = 0.04 for counselling-occupational therapy (superscript ‘a’ in Table 1), *p* = 0.02 for nursing-occupational therapy^b^). For anxiety scores, nursing students had a higher level than occupational therapy students and social work students (*p* = 0.04 for nursing-occupational therapy^c^, *p* = 0.03 for nursing-social work^d^). For community attitudes scores, occupational therapy students marked a lower level than all the other three student groups: occupational therapy students believed that their peers do not see mental health problems negatively (*p* = 0.01 for occupational therapy-counselling^e^, *p* < 0.01 for occupational therapy-nursing^f^ and occupational therapy-social work^g^). Lastly, nursing students demonstrated a higher level of self-reflected shame than counselling students: nursing students worried about their own reputation in relation to their family’s mental health problems more than counselling students^h^ (*p* < 0.01).

### Predictors of Mental Health Problems

To identify significant predictors of mental health problems in each group and the effect sizes, multiple regression analyses were conducted (Table 2). First, age was entered to statistically adjust for its effects on mental health problems (see Table 3 in the Appendix for our correlation table). Second, data for mental health problems, mental health shame and self-compassion were inputted. Adjusted coefficient of determination (adj. *R*^2^) was reported. Multicollinearity was of no concern (variance inflation factor < 10).

**Table 2:** Multiple regression: mental health shame and self-compassion to mental health problems among counselling, occupational therapy, nursing and social work students

	Counselling					Occupational therapy					Nursing					Social work				
	*B*	SE_B_	*β*	95% CI (lower, upper)		*B*	SE_B_	*β*	95% CI (lower, upper)		*B*	SE_B_	*β*	95% CI (lower, upper)		*B*	SE_B_	*β*	95% CI (lower, upper)	
Step 1																				
Age	− 0.77	0.37	− 0.24	− 1.51	− 0.02	− 0.35	0.35	− 0.12	− 1.05	0.34	− 0.76	0.41	− 0.19	− 1.57	0.04	− 0.48	0.22	− 0.21	− 0.91	− 0.04
Step 2																				
Age	− 0.36	0.34	− 0.11	− 1.04	0.33	− 0.09	0.29	− 0.03	− 0.67	0.49	− 0.26	0.27	− 0.06	− 0.79	0.27	− 0.13	0.23	− 0.06	− 0.59	0.34
Community attitudes	− 1.19	1.22	− 0.13	− 3.64	1.26	0.30	1.01	0.03	− 1.73	2.32	1.44	1.06	0.14	− 0.67	3.55	− 0.27	0.88	− 0.04	− 2.02	1.47
Family attitudes	− 2.01	1.61	− 0.24	− 5.23	1.22	0.10	1.10	0.01	− 2.10	2.30	0.68	1.09	0.07	− 1.49	2.85	− 1.13	0.91	− 0.15	− 2.94	0.68
Community external shame	0.89	0.94	0.15	− 0.99	2.77	0.02	0.71	< 0.001	− 1.38	1.43	− 0.16	0.76	− 0.02	− 1.66	1.35	0.44	0.73	0.08	− 1.00	1.88
Family external shame	1.83	1.59	0.24	− 1.35	5.00	1.89	0.89	0.24*	0.11	3.67	0.83	0.88	0.12	− 0.91	2.57	0.73	0.88	0.11	− 1.01	2.47
Internal shame	1.64	0.69	0.32*	0.27	3.02	0.53	0.71	0.10	− 0.88	1.94	0.83	0.57	0.13	− 0.30	1.95	0.59	0.61	0.12	− 0.62	1.79
Family-reflected shame	− 0.12	0.69	− 0.02	− 1.51	1.27	0.12	0.61	0.02	− 1.11	1.34	0.13	0.48	0.02	− 0.82	1.09	− 0.20	0.54	− 0.05	− 1.27	0.87
Self-reflected shame	− 0.14	0.87	− 0.02	− 1.89	1.60	0.74	0.54	0.13	− 0.35	1.83	0.37	0.48	0.05	− 0.59	1.32	0.99	0.57	0.18	− 0.15	2.12
Self-compassion	− 15.59	4.50	− 0.39***	− 24.59	− 6.59	− 21.06	3.51	− 0.54***	− 28.07	− 14.06	− 26.98	3.73	− 0.56***	− 34.38	− 19.58	− 8.90	3.58	− 0.29*	− 16.00	− 1.79

*B*, unstandardised regression coefficient; *SE*_*B*_, standard error of the coefficient; *β*, standardised coefficient; *CI*, confidence interval. *p < 0.05; ***p < 0.001

Self-compassion was the strongest or only predictor of mental health problems in all groups. The effects were largest in nursing students (51%), followed by occupational therapy (40%), counselling (21%) and social work (12%). In addition to self-compassion, internal shame was a significant predictor among counselling students, and family external shame was in occupational therapy students. No other predictors than self-compassion were identified in nursing and social work students.

## Discussion

Poor mental health of healthcare students is a cause of concern for many universities; however, understanding of their mental health remains to be refined. This study aimed to compare the levels of and relationships between mental health problems, mental health shame and self-compassion among counselling, occupational therapy, nursing and social work students. Our MANOVA revealed that (1) counselling and nursing students were more depressed than occupational therapy students, (2) nursing students were more anxious than occupational therapy and social work students, (3) occupational therapy students had more positive community attitudes than the rest and (4) nursing students worried about their own reputation in relation to their family’s mental health issues more than counselling students. Moreover, our regression analyses identified that (5) self-compassion was the strongest predictor of mental health in all groups; however, (6) the effect sizes were the largest in nursing students and smallest in social work students.

There are a number of possibilities which could explain these findings. Concerning mental health status among nursing students has been reported, relating to depression, anxiety and worries, often associated with their intensive practice experience (Li & Hasson, 2020). A recent study (Mills et al., 2020) further uncovered that nursing students juggle competing demands on their physical capabilities, personal resources, income and time. In their practical training, nursing students often experience difficult situations such as a death of a patient for the first time as a professional caregiver (Terry & Carroll, 2008). Moreover, nursing students are more at risk of experiencing bullying, including verbal abuse from patients and hospital visitors (Timm, 2014), whilst on clinical placement (Minton et al., 2018). Additionally, an irregular shift work pattern of nursing students on placement negatively impacts their mental health (Nea et al., 2018), which has been exacerbated during the pandemic (Propper et al., 2020). Moreover, many nursing students fail to seek out help, related to the guilt for self-care and fear of unsuitability to be a professional nurse (Kotera, 2021). Although these explanations cannot be a conclusive interpretation of our findings, it is likely that a combination of those challenging factors may put nursing students more at risk of mental health problems.

Contrast to nursing students may be occupational therapy students, who demonstrated still challenging yet better mental health status than nursing students. One explanatory factor may be that occupational therapy students’ placements and professional roles are often to enable patients with long-term conditions to live independently (Diamant et al., 2018). Occupational therapy is based on the bio-psychosocial framework to deliver person-centred interventions across the spectrum of care systems (Royal College of Occupational Therapists, 2019). Role-emerging placements, where there is no established occupational therapist role, are also increasingly being offered to occupational therapy students, augmenting students’ autonomy and self-directedness (Dancza et al., 2019). These unique features of the placement may help explain the differences in the level of mental health between occupational therapy students and nursing students.

Depression among counselling students may be attributable to their role function. Early career counsellors, including counselling students, are at an increased risk of burnout and depressive disorders (Kotera et al., 2021d; Simpson et al., 2019). Moreover, counselling treats mental health problems directly; therefore, the mental health impact can be large: for example, studying and treating people with depression can make them more vulnerable to depression (Yang & Hayes, 2020). Although self-reflection has been emphasised in counselling education (Quality Assurance Agency for Higher Education, 2013), help-seeking for mental health issues in this student group remains to be improved (Kotera et al., 2019a).

The high level of self-reflected shame (i.e. worrying about their own reputation in relation to their family’s mental health issues) in nursing students was a novel finding from this study. A strong professional identity among nursing students may help explain their critical scrutiny on themselves (Kotera et al., 2021a, 2021b). Indeed, nursing students are educated to be caring towards patients with mental illness; however, they preferred not to disclose their own mental health condition because of shame (Chang et al., 2017). This strong distinction between self and others may explain the high self-reflected shame in nursing students. Contrarily, counselling students are often aware of malleability of mental health resources (Lohoff, 2010), which may help explain their low level of self-reflected shame.

Lastly, our regression analyses indicate self-compassion as the strongest predictor of mental health problems across all groups in this study. Nursing students were found to exhibit the most pronounced effect of self-compassion on mental health, in line with previous findings (Kotera et al., 2021a, 2021b). Compassion is embedded in the principles of the professional body regulating UK nursing courses (Nursing and Midwifery Council, 2014) and forms a core part of UK nursing education (Durkin et al., 2018). A strong curricular emphasis on compassion and self-care could explain the large effect self-compassion has on the mental health of nursing students.

Although self-compassion is not yet included in any of the other disciplines’ codes of conduct or qualifying regulations, the positive psychological attribute of resilience is common to them all (British Association for Counselling & Psychotherapy, 2018; British Association of Social Workers, 2018; Royal College of Occupational Therapists, 2019). Resilience is strongly related to self-compassion (Bluth et al., 2018); however, this relationship was not found in social work students (Kotera et al., 2019b). This difference may explain the smaller effect of self-compassion on mental health for social work students in this study. As self-criticism has previously been identified as a mental health risk factor for social work students (Kotera et al., 2018), it is also likely that self-criticism is inhibiting the protective effect of self-compassion for this population (Kotera et al., 2021h). One relevant factor may be that social work attracts ‘wounded healers’, who have histories of hurt (Macfarlane, 2020). Many social work students have had adverse childhood experience, which later became a source of motivation for them to study social work (Thomas, 2016). Another factor may be that social workers support and advocate the most vulnerable people in the society; therefore, their work contexts are diverse, including less clinically controlled settings (Johnson et al., 2021). These factors may contribute to activation of self-criticism, which can help explain the smallest effect size of self-compassion on mental health in social work students.

The moderate effect of self-compassion on mental health was observed for counselling students. These students will likely have an awareness of self-compassion as a component of multiple third wave therapies (Ashworth et al., 2017), have experience of practicing self-compassion and compassion towards others (Beaumont et al., 2016) and consequently, benefit from the protective effect of self-compassion on mental health. Internal shame was found only to affect the mental health of counselling students. An important component of all counselling courses leading to professional registration is the requirement to undergo self-examination and reflection (British Association for Counselling & Psychotherapy, 2022a; Quality Assurance Agency for Higher Education, 2013), including supervision and, for many providers, a set amount of personal therapy (British Association for Counselling & Psychotherapy, 2022b). Students are expected to become aware of aspects of themselves that it may be difficult to encounter and to reconcile (Murphy et al., 2018). Unresolved conflict and ongoing self-examination may therefore have elevated internal shame for counselling students.

Taken together, self-compassion being the strongest predictor of mental health in all healthcare student groups suggests that these students may benefit from undertaking self-compassion training, consistent with previous research (Khorami et al., 2016). However, the difference of the effect sizes may inform how to implement self-compassion training in each discipline. For nursing, occupational therapy and counselling students, who demonstrated large to medium effects, self-compassion training may show effects by directly incorporating into their curriculum (Beaumont et al., 2017). However, for social work students, who demonstrated the smallest effect size, self-awareness practices can be introduced to them to reflect their own mental health, in order to maximise the effects of self-compassion training, mitigating a sense of guilt from caring for themselves (Kotera, 2021). Future research needs to explore how self-compassion training is implemented to maximise the positive impact on the mental health of healthcare students.

Whilst this study offered helpful insights, several limitations need to be noted. First, all samples were collected from one university in the UK through opportunity sampling, and students in other healthcare subjects were not recruited: a wider recruitment is needed in future research. Although the scales in this study were commonly used in the field, the accuracy remains to be refined (e.g. SCS-SF (Kotera & Sheffield, 2020)). Additionally, response biases might have been present as these were self-report measures (Kotera et al., 2020c). Lastly, the causality of the relationships identified in this study was not evaluated. Longitudinal data would help assess the temporal patterning of these relationships.

## Conclusion

Healthcare students suffer from high rates of mental health problems. Shame towards mental health problems and self-compassion have been identified as key factors for the mental health in this student group; however, differences of these constructs across different healthcare disciplines remained to be evaluated. We identified the differences in the levels and relationships of these mental health constructs between counselling, occupational therapy, social work and nursing students. Nursing students had poorer mental health, and occupational therapy students had the least negative attitudes towards mental health problems. Self-compassion was identified as the strongest predictor of mental health in all four groups; however, the effect size was largest in nursing and smallest in social work students. The difference in the effect sizes may inform the implementation of self-compassion training in their curriculum. Our findings will help identify effective strategies to protect the mental health of healthcare students in each discipline.

## Data Availability

The datasets generated during and/or analysed during the current study are available from the corresponding author on reasonable request.

## Appendix

Table 3

**Table 3:** Correlation table for demographics and study variables in all healthcare students

		1	2	3	4	5	6	7	8	9	10	11	12	13
1	Gender (1 = M, 2 = F)	-												
2	Age	− 0.052	-											
3	Depression	− 0.003	− 0.150**	-										
4	Anxiety	0.064	− 0.195**	0.707**	-									
5	Stress	0.068	− 0.160**	0.773**	0.754**	-								
6	Community attitudes	− 0.074	0.199**	0.180**	0.128*	0.182**	-							
7	Family attitudes	− 0.059	0.053	0.279**	0.167**	0.216**	0.512**	-						
8	Community external shame	− 0.074	0.088	0.347**	0.272**	0.297**	0.577**	0.421**	-					
9	Family external shame	− 0.077	0.069	0.390**	0.287**	0.298**	0.342**	0.699**	0.554**	-				
10	Internal shame	0.067	− 0.209**	0.484**	0.352**	0.447**	0.117*	0.175**	0.415**	0.293**	-			
11	Family-reflected shame	− 0.107*	− 0.019	0.332**	0.234**	0.285**	0.341**	0.391**	0.524**	0.503**	0.512**	-		
12	Self-reflected shame	− 0.027	− 0.094	0.248**	0.219**	0.217**	0.245**	0.283**	0.281**	0.254**	0.278**	0.397**	-	
13	Self-compassion	− 0.099	0.195**	− 0.524**	− 0.460**	− 0.585**	− 0.110*	− 0.169**	− 0.199**	− 0.219**	− 0.395**	− 0.212**	− 0.143**	-

**p* < .05, ***p* < .01
